# Maxillary Sinus Augmentation with Autogenous Tooth Grafting Material: A Systematic Review

**DOI:** 10.3390/biomimetics9090518

**Published:** 2024-08-29

**Authors:** Diba Ghodsian, Sofía D’Jesús, Luis Sánchez-Labrador, Carlos Manuel Cobo-Vázquez, Jorge Cortés-Bretón Brinkmann, José María Martínez-González, Cristina Meniz-García

**Affiliations:** Department of Dental Clinical Specialties, Faculty of Dentistry, Complutense University of Madrid, 28040 Madrid, Spain; dibaghod@ucm.es (D.G.); sdjesus@ucm.es (S.D.); luissanc@ucm.es (L.S.-L.); cmcobo@ucm.es (C.M.C.-V.); jmargo@ucm.es (J.M.M.-G.); cmenizga@ucm.es (C.M.-G.)

**Keywords:** autogenous tooth graft material, maxillary sinus floor elevation, histomorphometry, biological complications, systematic review

## Abstract

The aim of this systematic review was to determine whether autogenous tooth grafting material (ATGM) is as safe and effective as other bone substitutes used for maxillary sinus augmentation procedures, evaluating histomorphometric and/or histological data, implant primary stability, associated complications and radiographic bone height measurements. An automated electronic search was conducted using four databases (Medline/PubMed, Scopus, Web of Science and Cochrane Library), supplemented by a manual search, to identify clinical human studies using particulate ATGM for the aforementioned procedure. The included studies had a sample size of at least four patients and were published before 31st July 2024. The Newcastle–Ottawa scale (NOS) and Joanna Briggs Institute (JBI) Critical Appraisal Checklist were used to assess the risk of bias in cohort studies and case series, respectively. Seven studies were included in the descriptive analysis, obtaining 128 participants (46.8% only treated with ATGM) and 192 placed implants. Due to the heterogeneity of the studies, meta-analysis could not be performed. The authors concluded that ATGM appears to be a feasible and safe alternative for maxillary sinus augmentation procedures. These results should be interpreted with caution due to the limited amount of scientific evidence on this topic and the heterogeneity between the included studies.

## 1. Introduction

Implant placement is currently a widely performed dental procedure for restoring the edentulous posterior maxilla, with a documented clinical success rate of above 90% after five years of follow-up [1], with its success reliant on multiple factors, including patient-related factors (systemic health, local factors and social history) and anatomical factors [2]. Tooth loss in the posterior maxillary region triggers the physiological process of bone resorption, as well as further maxillary sinus pneumatization, resulting in an insufficient residual bone height for implant placement, requiring additional regenerative surgical procedures, such as sinus lift augmentation procedures [1].

Maxillary sinus augmentation by bone was first described by Tatum in 1977, and then published by Boyne and James in 1980 [3]. It is a well-documented surgical technique, supported by strong scientific evidence, and it is one of the therapeutic options for placing implants in atrophic posterior maxilla (particularly when the residual bone height is less than 5 mm) [4].

Bone regeneration is accomplished by elevating the Schneiderian membrane and creating a virtual cavity to be filled with compact bone substitute material; this will ultimately provide sufficient bone of acceptable height and quality for implant placement and subsequent prosthetic restoration. In order to protect the regenerated region and avoid graft material displacement, a resorbable membrane may be placed prior to suturing [5,6,7,8]. A wide variety of bone substitutes have been studied, such as xenograft, allograft and synthetic biomaterials. Whilst autogenous bone is considered the gold standard in bone regeneration (due to its osteogenic, osteoconductive and osteoinductive properties), it presents certain disadvantages: for instance, it requires an additional and/or more complex surgical intervention to obtain the graft from a donor site, it offers a limited amount of graft material, and has a high resorption rate (of around 50% in particulate form, as reported by certain authors) [9,10].

Due to these drawbacks, Kim et al. [11] proposed autogenous teeth as bone graft material. This bone substitute reduces postoperative morbidity, has osteoconductive and osteoinductive properties [12], reduces treatment costs [13], and has a chemical composition similar to human bone: 65% inorganic component (including hydroxyapatite, tricalcium phosphate, octacalcium phosphate, amorphous, calcium phosphate), 35% organic component, mainly type I collagen and non-collagen proteins (including bone morphogenic proteins, bone sialoprotein, osteopontin, osteonectin, among others) and water [13,14,15]. Furthermore, its use is well accepted by patients and shows promising results in regenerative procedures [13] and adequate behavior in lateral access sinus lift procedures with 20 years follow-up [16].

Additionally, no clear consensus exists regarding which of the available bone substitute material is more appropriate for maxillary sinus augmentation procedures [17,18]. Although there are multiple published systematic reviews on sinus lift techniques [19,20,21], to the authors’ knowledge, there are no published systematic reviews that evaluate the use of autogenous tooth grafting material (ATGM) for maxillary sinus augmentation. Therefore, the aim of this systematic review is to analyze the efficacy of ATGM and compare it with other bone substitutes, in terms of histomorphometric and/or histological data, implant primary stability, associated complications and radiographic bone height measurements.

## 2. Materials and Methods

This systematic review followed the PRISMA (Preferred Reporting Items for Systematic Review and Meta-Analyses) statement [22] and was registered in the PROSPERO International Prospective Register of Systematic Reviews (Centre for Reviews and Dissemination, University of York, National Institute for Health Research, United Kingdom; Reg. no. CRD42023387027).

This study aimed to answer the following PICOS question (Population, Intervention, Comparison, Outcome and Study Design) [23]: “In partially edentulous patients with atrophic posterior maxillae to be restored with dental implants, is ATGM appropriate for maxillary sinus augmentation procedures, in terms of histomorphometry, histology, primary implant stability, complications and radiographic bone height measurements, when compared to other bone regeneration materials?”, defined as follows:

- Population: Adult patients with atrophic edentulous posterior maxillae to be restored with dental implants, with at least one donor tooth (of hopeless periodontal prognosis/impacted).
- Intervention: Maxillary sinus augmentation being grafted with ATGM as regeneration material.
- Comparison: Maxillary sinus augmentation using other types of grafting materials: autogenous bone, xenografts, allografts or synthetic grafts.
- Outcome: The main outcome is the histomorphometric and/or histological data (percentage of vital bone, connective tissue and residual graft). Secondary outcomes are primary implant stability, intra- and post-operative associated complications and radiographic bone height measurements.
- Study Design: Clinical studies (clinical trials, cohort studies and case series).

### 2.1. Eligibility Criteria

#### 2.1.1. Inclusion Criteria

- Clinical studies: clinical trials, cohort studies and case series.
- Sample size of at least 4 participants.
- Studies where lateral access maxillary sinus augmentation was performed using ATGM, exclusively or mixed with other biomaterials.
- Studies evaluating at least one of the proposed outcomes: histomorphometric data, histological data, intra- and post-surgical complications, bone height measurements, and/or primary implant stability.
- Papers published until 31st July 2024 (included).

#### 2.1.2. Exclusion Criteria

- Case reports.
- Animal and in vitro studies.
- Studies in which ATGM is used for different surgical procedures.
- Studies evaluating transalveolar sinus lift procedures.

### 2.2. Sources and Search Strategy

An automated search was carried out in the following databases: (1) MEDLINE (via PubMed), (2) Web of Science, (3) Cochrane Central Register of Controlled Trials and (4) Scopus. The filters and limits used were human studies and publication date up to and including 31st July 2024. The search strategy combined MeSH (Medical Subject Headings) terms and free terms using the following formula: [Material AND Intervention].

Material: “dentin” OR “particulate dentin” OR “dentin matrix” OR “demineralized dentin matrix” OR “autologous tooth” OR “tooth graft” OR “autogenous tooth bone graft”.

Intervention: “maxillary sinus” OR “sinus lift” OR “maxillary sinus lift” OR “sinus augmentation” OR “maxillary sinus augmentation” OR “sinus elevation” OR “maxillary sinus elevation” OR “maxillary sinus floor elevation” OR “lateral sinus lift” OR “lateral sinus elevation” OR “open sinus elevation” OR “lateral sinus lift” OR “open sinus lift”.

The manual search was conducted in the following Oral Surgery and Implantology journals: *Oral and Maxillofacial Surgery Clinics of North America*, *International Journal of Oral Maxillofacial Surgery*, *International Journal of Oral and Maxillofacial Implants*, *International Journal of Oral Surgery*, *Journal of Cranio-Maxillofacial Surgery*, *Journal of Dentistry*, *Clinical Oral Implants Research*, *Clinical Implant Dentistry and Related Research*, *Implant Dentistry*, *European Journal of Oral Implants* and *Journal of Oral and Maxillofacial Surgery*. The references of the retrieved papers and review articles on this review’s subject were also revised.

### 2.3. Study Selection and Screening Methods

Two reviewers (D.G.N and S.D.J) independently screened the titles and abstracts of the identified articles. The reviewers then read the full manuscripts of studies that complied with the eligibility criteria, as well as those with insufficient data in the title and abstract, before making the final selection. If any disagreement arose during this process, it would be solved by a third author (L.S.L). For the detection of duplicate references, the Zotero tool was used (Center for History and New Media, University George Mason, Virginia, United States). Inter-reviewer reliability during the search and article selection process was calculated to obtain a percentage of agreement and kappa correlation coefficient.

### 2.4. Data Collection and Items

The primary outcomes in this review were histomorphometric and/or histological data, considering the percentages of vital bone, connective tissue and residual graft material at re-entry for implant placement. The secondary outcomes were implant primary stability (ISQ values), intra- and postoperative complications associated and radiographic bone height measurements. Additionally, descriptive information on ATGM and the clinical results of the procedure were included (new bone formation, re-entry timings and marginal bone loss after implant placement). Data extraction was carried out and summarized by charting the information on each outcome independently; when the information provided was incomplete, the third author was involved in the decision process.

Extracted data were as follows: author, journal and year of publication, study design, graft material used, number of patients, average age, gender, systemic conditions, total number of sinus lifts, total number of implants placed, residual bone height (initial), new bone volume, final bone height, selection criteria for the donor teeth, re-entry time, implant primary stability (ISQ recorded at the moment of implant placement), implant placement protocol (simultaneous or delayed), marginal bone loss, follow-up, associated complications, histomorphometric and histological data, and ATGM characteristics.

### 2.5. Risk of Bias Assessment

Risk of bias assessment of cohort studies was performed using The Newcastle–Ottawa scale (NOS) [24]. This scale includes 3 domains: selection of the study group, comparability between participants and the studied outcome. Each study received a maximum of 9 points, individually. For analyzing the quality of case series, the Joanna Briggs Institute (JBI) Critical Appraisal Checklist for Case Series was used, which consists of 10 questions that consider the paper’s methodological aspects, as well as the report of results [25].

### 2.6. Statistical Analysis

Due to the heterogeneity of the included articles, meta-analysis could not be performed. Therefore, only qualitative analysis was performed for each outcome.

## 3. Results

### 3.1. Study Selection

A total of seven hundred seventy-seven publications were identified, out of which seven hundred seventy-two papers were obtained from the automated search and five additional articles by manual search. Duplicates and triplicates were discarded with the Zotero tool. After the initial screening of the remaining publications, twelve papers were selected for full-text analysis, as shown in Table 1, where reasons for the exclusion of the five discarded articles are described. A total of seven papers, two of them belonging to the same study by Minetti et al. [26,27], were included in this systematic review (96.15% of agreement between reviewers; kappa index = 0.89). Five of the included papers registered the primary outcome [26,27,28,29,30] and two of them registered at least one of the secondary outcomes [2,31]. Due to the scarce literature, non-comparative studies were included in the present systematic review.

**Figure 1:**
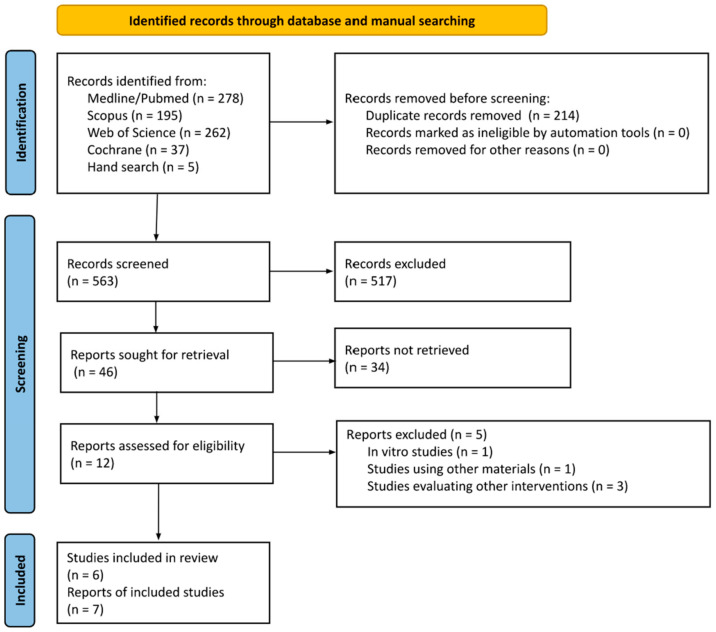
PRISMA 2020 flow chart depicting the search strategy and article selection process.

Figure 1 represents the searching and selection process that was carried out, following the PRISMA 2020 guidelines [22].

### 3.2. Study Characteristics

Out of the seven included studies, five were case series and two were cohort studies (one prospective and one retrospective). Only two comparative studies employed ATGM in the experimental group and compared the results obtained with other biomaterials. In the prospective cohort study, the control group was treated with Bio-Oss© (Geistlich Pharma AG, Wolhusen, Switzerland) [28], while the retrospective cohort study had three groups: one experimental group (ATGM) and two positive control groups (one treated with demineralized freeze-dried bone allograft and another with Bio-Oss^®^) [2].

The remaining five studies were case series. In three of them, only ATGM was used [26,27,29]; however, the other two studies used a combination of ATGM with other materials: platelet-rich plasma (PRP) [31], autogenous bone and/or xenograft [30]. Table 2 and Table 3 contain the main characteristics of the included studies. Overall, the studies showed great heterogeneity in terms of the methodology and the reporting of outcomes.

### 3.3. Quality Assessment of Individual Studies

Table 4 depicts the risk-of-bias scores for the included cohort studies; both Jun et al. [28] and Jeong et al. [2] studies demonstrated a low risk of bias. Table 5 represents the case series assessment process by using the JBI Critical Appraisal Tool, resulting in a lower risk of bias for four studies [26,27,29,31], which presented affirmative answers to almost all of the questions of the tool. The study by Pohl et al. [30] resulted in an intermediate risk of bias.

### 3.4. Synthesis of Results

#### 3.4.1. Patient Characteristics

The two cohort studies [2,28] included sixty-four patients, whilst the five case series [26,27,29,30,31] included another sixty-four patients, obtaining a total of one hundred twenty-eight participants; sixty of them were only treated with ATGM, twenty-seven were treated with a mixture of ATGM and another biomaterial, two were treated with a mixture of ATGM and more than one biomaterial, twenty-eight were treated with xenograft material, and eleven were treated with allograft material. Regarding demographic data, the mean ages of the patients varied from 40 to 64 years old and the gender distribution was 54% males and 46% females; however, two studies failed to report this information [2,29]. Only one study [30] did not report the participants’ systemic condition, and the other six performed maxillary sinus floor elevations in ASA I and II patients (Table 2).

#### 3.4.2. Histomorphometric Data

Three of the included studies, two case series [26,27] and the prospective cohort study [28] carried out histomorphometric analysis (Table 6).

The only comparative study [28], which performed histomorphometry, showed that the percentage of newly formed vital bone after 4 months was greater when employing ATGM (31.07%), whereas connective tissue (39.93%) and residual dentin graft (29%) were lower in comparison to the control group treated with Bio-Oss© (26.49%, 42.38% and 31,12%, respectively); the differences were not statistically significant for any of the three variables (*p* = 0.556). However, these authors also registered the mean thickness of the osteoid tissue, being 8.35 μm for the Bio-Oss© group and 13.12 μm for the group treated with ATGM; in this case, there was a statistically significant difference (*p* = 0.025).

The remaining two case series [26,27] were both published by Minetti et al.; however, some differences in methodology were detected. One of the studies carried out the histomorphometric analysis at 4 months and reported 36.28% of new vital bone volume and 14.61% of residual dentin [26].

The last case series by Minetti et al. [27] carried out the histomorphometric analysis at 6 months and reported identical values for bone volume (BV) and residual dentin percentages as the previous study. This study included a total of 19 additional patients in comparison to the previous study [26], and the authors also reported the vital bone percentage excluding medullary tissue and residual graft, obtaining a value of 21.51% after 6 months [27].

#### 3.4.3. Histological Data

Among the seven reviewed studies, five of them registered histological data [26,27,28,29,30]. In all of them, bone formation over the ATGM was detected, including bone cells (osteoblasts, osteoclasts [28,30] and even osteocytes [26] and bone formed de novo; moreover, two studies reported the presence of bridge formation between graft materials [28,30]. Two studies pointed out the formation of medullary space composed of well-vascularized connective tissue in the newly formed bone [26,28].

Pohl et al. [30] performed seven biopsies in their study. They highlighted the presence of bone formation activity surrounding the dentin portion of the graft as well as in the enamel portion. The amount of newly formed bone covering the graft was similar in both portions.

Minetti et al. reported that, in some cases, dentin granules appeared to be completely incorporated within woven bone and surrounded by a layer of developing osteoid tissue; however, some coronal granules were surrounded by fibrous tissue [26].

Taking into account all the analyzed patients in both studies conducted by Minetti et al. [26,27], no infectious or inflammatory reactions adjacent to the graft material were reported in any of the 27 patients.

#### 3.4.4. Complications

Two of the included studies reported complications [29,31]; three of them did not report any complications [26,27,30] and two of them did not register this variable [2,28]. A total of one hundred ninety-two dental implants were placed, eight (4.16%) reported complications (intra- or post-operative) and three of them failed (1.56% failure rate). Considering complications of any kind, two of the implants were delayed [29] and the remaining six were placed simultaneously [31]. With regard to the failed implants, one of them was delayed [27] and the other two were placed simultaneously [31].

The overall complication rate of the maxillary sinus floor elevations was 5.72%. The survival rate of the implants varied from 97.01% and 100%, with follow-up periods of 6–60 months. The results of this variable are summarized in Table 7. The most common complication during the sinus lift procedure was a perforation of the Schneiderian membrane.

#### 3.4.5. Implant Stability

Only one of the seven studies reported ISQ values at placement [28]. None of them registered insertion torque. The aforementioned study measured the ISQ values of 57 delayed placement implants (re-entry after 4 months), 28 of them were placed in a control group in which the sinus lift was grafted with Bio-Oss^®^, while the other 29 from experimental group were grafted with ATGM [28].

The device used to measure ISQ values was Osstell Mentor© (Goteborg, Sweden), and the mean value obtained was 64.92 when ATGM was used; in contrast, the value obtained for implants placed in control group was 70.59 (28 OII using Bio-Oss), without statistically significant differences.

#### 3.4.6. Radiographic Bone Height Measurements

Five of the studies registered bone height measurements [2,27,28,29,31]. Three of them took radiographic measurements on CBCT [27,28,29] and two of them [2,31] used panoramic radiographs. The results are summarized in Table 3.

#### 3.4.7. Surgical Procedure and Tooth Preparation

Two publications reported the number of maxillary sinus augmentation performed [2,30]. A total of 192 dental implants were placed (82 were placed in patients treated only with ATGM), the study by Minetti et al. [26] was the only one that did not report any data on implant placement. Overall, out of 192 dental implants, 71 dental implants were placed simultaneously and 121 dental implants were delayed (Table 2 and Table 3).

Only Minetti et al. [26,27] Pohl et al. [30] reported in their studies the device employed for the preparation of ATGM: the Tooth Transformer (TT^®^) [26,27] and Medos Austria’s manual mill [30]. The tooth preparation procedure was described in six studies [26,27,28,29,30,31]; four of them specified the tooth materials and tissues removed during the preparation process of the biomaterial [26,27,28,29]. Only Jun et al. [28] indicated the particle size of the biomaterial after processing (0.5–1 mm). The treatment of the dentin was described in six studies [26,27,28,29,30,31], although it was different in each one of them.

#### 3.4.8. Re-Entry

Among the seven selected studies, two of them did not describe re-entry [2,31]; in one of them, all the implants were placed simultaneously during the maxillary sinus augmentation procedure [31], whilst in the other one, the timing of implant placement was not reported [2]. In the remaining five studies, the re-entry ranged from 3 to 6 months (Table 3).

## 4. Discussion

The aim of this systematic review was to analyze the use of ATGM in lateral access maxillary sinus augmentation, in terms of histomorphometric/histological data and clinical variables (implant stability, associated complications and bone height measurements). A total of seven studies (five case series and two cohort studies) have been included.

Besides an acceptable resorption rate, an ideal bone regeneration biomaterial should also have osteogenesis, osteoconduction and osteoinduction. As of today, only autologous bone fulfills those characteristics. However, it has limited availability and requires additional surgeries, which is linked to higher post-operative morbidity [13,37]. Due to these drawbacks, the use of ATGM has gradually extended since Kim et al. [11] described its use for guided bone regeneration (GBR) procedures simultaneous to implant placement. Numerous papers have been published since then using ATGM alone or in combination with other biomaterials for GBR [11,38,39], alveolar ridge preservation [40,41,42], immediate post-extractive implants [43], bone defect regeneration and following the extraction of the impacted teeth [44,45,46]. Furthermore, this material can be used in a particulated form or blocks [47], although particulate is more widespread and has more scientific evidence. ATGM has shown good clinical and radiographic behavior, potentially due to its chemical similarity to autologous bone, which makes it biocompatible and bioactive. Additionally, it requires less time and is more conservative to be obtained in comparison to autologous bone, and it is also well-accepted by patients [14,37,48]. However, ATGM needs a donor tooth, and it can only be used in patients with remaining teeth that have a hopeless prognosis and those with an atrophic posterior maxilla (less than 5 mm of bone height) but with adequate width.

Regarding osteoinduction, it seems the organic component of ATGM plays a fundamental role in this process in human and animal models. The presence of calcium and phosphate salts allows it to be used as scaffolding for the osteoclasts and osteoblasts of the recipient surface, showing osteoconductive properties. Enamel is a highly mineralized tissue, leading to increased osteoconductive properties when it is included in the graft [49,50].

Histomorphometric and histological data

Considering histomorphometric data, in this systematic review, BV percentage varied from 21.51 to 36.28% [26,27,28] when only ATGM was used after 4–6 months of sinus lift augmentation. In this regard, ATGM has shown higher BV quantities in comparison to xenografts [51].

Moreover, ATGM presented lower quantities of residual graft material, potentially revealing a better resorption rate [26,27,28]. Due to heterogeneity amongst the included studies, it was impossible to obtain a valid conclusion regarding whether ATGM present improved resorption rates and remodeling ability compared to other grafting materials. However, multiple comparative animal studies [52,53,54] reported significantly higher bone formation when using ATGM [53].

A low complication rate of 5.72% was obtained from the seven studies included in this systematic review. Kim et al. [55] reported that the most frequent complications were as follows: a perforation of the Schneiderian membrane (60%), post-operative infections (21%) and bleeding (9%). It was not specified whether these numbers varied depending on the biomaterial used for the procedure. In compliance with these data, the most frequent complication reported in the studies included in this systematic review was a perforation of the Schneiderian membrane. Thus, it can be assumed that ATGM does not present a higher risk of complications compared to other biomaterials. Nevertheless, to the authors’ knowledge, the availability of the literature is scarce, which did not allow a comparison of ATGM with other biomaterials in terms of complications rates. The survival rate of the present systematic review varied from 97.5% at 1 year (40 OII in total) [27], 97.01% at 4.5 years (67 OII in total) [31] and 100% at 5 years post-operation (15 OII in total) [30]. Overall, a total of 192 OII were placed, 4.16% being linked to any kind of intra- or post-operative complication and 1.56% failed. These data are similar to the failure rate when using other grafting materials.

Finally, this systematic review has several limitations, mainly related to the lack of the available literature; hence, the included studies are five case series and two cohort studies (one prospective and one retrospective), resulting in a higher risk of bias. Thus, the results should be interpreted with caution. Moreover, the cases must comply with some requirements to be performed (patients with at least one donor tooth, impacted or with impossible periodontal prognosis to be used in the maxillary sinus augmentation procedure), and it also requires a tooth processing device to obtain the ATGM. The amount of obtained biomaterial is sometimes not enough to perform a lateral access sinus lift procedure, so two of the included studies mixed the ATGM with other biomaterials, thus representing a considerable limitation of the present systematic review.

Another source of bias is the relatively small sample size for histomorphometric analysis. The studies reported different methods of tooth processing and compared different biomaterials; in some cases, the ATGM was mixed with other graft materials and no distinction was made in the results. In this regard, further clinical studies with standardized protocols are required to obtain more reliable results.

## 5. Conclusions

Despite the limitations of this systematic review, it can be concluded that ATGM is a feasible and safe alternative grafting material for maxillary sinus augmentation procedures, showing good clinical and histological/histomorphometrical results in comparison with autogenous bone and other biomaterials (bovine xenografts or allografts). Its biocompatibility and bioactivity is similar to autogenous bone, with low complication rates and is less costly when compared to different bone substitutes.

However, these results should be interpreted with caution due to the nature of the included studies and the heterogeneity in the methodology of the available scientific studies. Therefore, further long-term clinical studies with a homogeneous methodology and larger samples are required in order to better understand ATGM behavior.

## Figures and Tables

**Table 1:** Articles selected for full text analysis.

Authors	Publication Year	Journal	Institution (Country)	Title	Inclusion/Exclusion	Reasons for Exclusion
Jun et al. [28]	2014	*J Adv Prosthodont*	NS (Korea)	A prospective study on the effectiveness of newly developed autogenous tooth bone graft material for sinus bone graft procedure	Included	NA
Jeong et al. [2]	2014	*Maxillofac Plast Reconstr Surg.*	Division of Oral and Maxillofacial Surgery, Department of Dentistry in Ajou University Hospital (Korea)	The Efficacy of the Graft Materials after Sinus Elevation: Retrospective Comparative Study Using Panoramic Radiography	Included	NA
Pohl et al. [30]	2016	*Int J Oral Maxillofac Implants*	Department for Oral and Maxillofacial Surgery, Medical University of Vienna (Austria)	A New Method Using Autogenous Impacted Third Molars for Sinus Augmentation to Enhance Implant Treatment: Case Series with Preliminary Results of an Open, Prospective Longitudinal Study	Included	NA
Fattouh et al. [29]	2018	*EDJ*	Department of Oral and Maxillofacial Surgery, Faculty of Oral and Dental Medicine, Cairo University (Egypt)	Clinical, radiographic and histological outcomes of sinus floor augmentation for delayed implant placement using autogenous fresh tooth graft	Included	NA
Minetti et al. [26]	2019	*BAOJ Dentistry*	NS (Italy)	Tooth Transformer: A New Method to Prepare Sinus Lift Autologous Toothgrafts. Histologic and Histomorphometric Analyses of 4 consecutive Clinical Cases	Included	NA
Minetti et al. [27]	2019	*Int J Growth Factors Stem Cells Dent*	4 private dental clinics (Czech Republic and Italy)	Autologous Tooth Graft for Maxillary Sinus Augmentation: A Multicenter Clinical Study	Included	NA
Ha et al. [31]	2019	*J Korean Dent Sci*	Department of Oral and Maxillofacial Surgery at Ulsan University Hospital (Korea)	Maxillary Sinus Floor Augmentation Using Autogenous Tooth Bone Graft in Combination with Platelet-Rich Plasma for Dental Implants: Case Series	Included	NA
Jeong et al. [32]	2011	*Implant Dent*	Dental Clinic of Chosun University and Seoul National University Bundang Hospital (Korea)	Clinical Study of Graft Materials Using Autogenous Teeth in Maxillary Sinus Augmentation	Excluded	Evaluates crestal approach in maxillary sinus augmentation
Kim et al. [33]	2013	*Implant Dent*	Department of Oral and Maxillofacial Surgery of Seoul National University, Bundang Hospital and Seoul In Dental Clinic (Korea)	Bone Grafts Using Autogenous Tooth Blocks: A Case Series	Excluded	Evaluates multiple regenerative procedures, with only three sinus lift cases
Kim et al. [34]	2014	*J Periodontal Implant Sci*	Seoul National University Bundang Hospital Dental Department (Korea)	Comparison of autogenous tooth bone graft and synthetic bone graft materials used for bone resorption around implants after crestal approach sinus lifting: a retrospective study	Excluded	Evaluates crestal approach in maxillary sinus augmentation
Kim et al. [35]	2016	*Springerplus*	NS (Korea)	Space maintenance in autogenous fresh demineralized tooth blocks with platelet-rich plasma for maxillary sinus bone formation: a prospective study	Excluded	Use of tooth blocks instead of particulate ATGM
Bono et al. [36]	2017	*J Appl Biomater Funct Mater*	Polytechnic of Milan (Italy)	Demineralized dentin and enamel matrices as suitable substrates for bone regeneration	Excluded	In vitro study

NS: not specified; NA: not applicable.

**Table 2:** Analyzing study design, biomaterials, age, gender, number of sinus lift procedures and implants placed.

Author, Year. Journal	Study Design	Other BM	Nº of Patients			Mean Age		Gender		Nº of Sinus Lift	Nº of OII		
			Total	ATGM	Comparison	ATGM	Comparison	Men	Women		Total	ATGM	Comparison
Jun et al., 2014. [28] *J Adv Prosthodont*.	Prospective cohort study	X	38	19	19	53.15	58.21	24	14	NR	57	29	28
Jeong et al., 2014. [2] *Maxillofac Plast Reconstr Surg*.	Retrospective cohort study	Al, X	26	6	20	NR	NC	NR		30	NR		
Pohl et al., 2016. [30] *Int J Oral Maxillofac Implants.*	Case series	AB and X	6	4 *, 2 **	NC	40	NC	1	5	9	15	9 *, 6 **	NC
Fattouh and Ali, 2018. [29] *EDJ.*	Case series	No	8	8	NC	NR	NC	NR		NR	13	13	NC
Minetti et al., 2019. [26] *BAOJ Dentistry*	Case series	No	4	4	NC	52,5	NC	3	1	NR	NR		
Minetti et al., 2019. [27] *Int J Growth Factors Stem Cells Dent*	Case series	No	23	23	NC	57,1	NC	9	14	NR	40	40	NC
Ha et al. 2019. [31] *J Korean Dent Sci.*	Case series	PRP	23	23 *	NC	53.78	NC	14	9	NR	67	67 *	NC

(*): autogenous tooth grafting mixed with one other biomaterial; (**): autogenous tooth bone graft mixed with more than one biomaterial; AB: autogenous bone; Al: allograft; ASA: classification system of the physic health by the American Society of Anesthesiologists; ATGM: autogenous tooth grafting material; BM: biomaterial; NC: no comparison group; NR: not reported; OII: osseointegrated dental implants; PRP: platelet-rich plasma; X: xenograft.

**Table 3:** Analyzing implant placement protocol, re-entry time and bone measurements.

Author, Year. Journal	Nº of OII Depending on the Placement Protocol		Re-Entry Time (Months)	Mean Pre-Surgery BH (mm)		BG (mm)		Final BH (mm)		Mean Marginal Bone Loss (mm per Year)	
	Simultaneous	Delayed		ATGM	Other BM	ATGM	Other BM	ATGM	Other GM	ATGM	Other BM
Jun et al., 2014. [28] *J Adv Prosthodont.*	0	57	4	3.12	3.17	10.45	10.73	13.56	13.90	NR	
Jeong et al., 2014. [2] *Maxillofac Plast Reconstr Surg.*	NR		NR	5.55	5.9	9.07	11.30	14.63	17.20	1.27	1.45
Pohl et al., 2016. [30] *Int J Oral Maxillofac Implants.*	4	11	4–10 months	NR		NR		NR		0.63	
Fattouh and Ali, 2018. [29] *EDJ.*	0	13	6	3		12.3		15.3		2.7	NC
Minetti et al., 2019. [26] *BAOJ Dentistry*	NR		4	NR		NR		NR		NR	
Minetti et al., 2019. [27] *Int J Growth Factors Stem Cells Dent*	0	40	6	5.22		9.50		14.72		NR	
Ha et al. 2019. [31] *J Korean Dent Sci.*	67	0	NRE	4.45		NR		NR		0.12	

ATGM: autogenous tooth bone graft; BG: bone gain; BH: bone height; BM: biomaterial; NC: no comparison group; NR: not reported; NRE: no re-entry; OII: osseointegrated dental implants.

**Table 4:** Risk of bias assessment of cohort studies using the Newcastle–Ottawa Scale.

Study	Selection				Comparability		Results			Total Score (above 8)
	S1	S2	S3	S4	C1	C2	R1	R2	R3	
Jun et al. 2014. [28]	**★**	**★**	**★**	**★**	**★**	**0**	**★**	**★**	**★**	8
Jeong et al. 2014. [2]	**★**	**★**	**★**	**★**	**★**	**0**	**★**	**★**	**★**	8

**Table 5:** Risk of bias assessment of case series using the Joanna Briggs Institute Critical Appraisal Tool.

Study	Pohl et al. 2016. [30]	Fattouh et al. 2018. [29]	Minetti et al. 2019. [26]	Minetti et al. 2019. [27]	Ha et al. 2019. [31]
1. Were there clear criteria for inclusion in the case series?	+	+	?		+
2. Was the condition measured in a standard, reliable way for all participants included in the case series?	?	+	?	+	+
3. Were valid methods used for identification of the condition for all participants included in the case series?	+	+	+		?
4. Did the case series have consecutive inclusion of participants	+	?	+	+	+
5. Did the case series have complete inclusion of participants?	+	?	+	+	+
6. Was there clear reporting of the demographics of the participants in the study?	+	−	+	+	+
7. Was there clear reporting of clinical information of the participants?	+	+	−	+	+
8. Were the outcomes or follow up results of cases clearly reported?	+	+	+	+	+
9. Was there clear reporting of the presenting site(s)/clinic(s) demographic information?	−	+	−	?	+
10. Was statistical analysis appropriate?	+	+	NA	+	−
Global evaluation	Included	Included	Included	Included	Included

+ = Yes; − = No; ? = Unclear; NA = Not applicable.

**Table 6:** Histomorphometric data of the included studies.

Author, Year. Journal	Re-Entry	% Vital Bone		% Connective		% Residual Graft	
		Only ATGM	Other BM	Only ATGM	Other BM	Only ATGM	Other BM
Jun et al., 2014. [28] *J Adv Prosthodont.*	4 months	31.07%	26.49%	39.93%	42.38%	29%	31.12%
Jeong et al., 2014. [2] *Maxillofac Plast Reconstr Surg.*	NR	NR	-	NR	-	NR	-
Pohl et al., 2016. [30] *Int J Oral Maxillofac Implants.*	4–10 months	NR	-	NR	-	NR	-
Fattouh and Ali, 2018. [29] *EDJ.*	6 months	NR	-	NR	-	NR	-
Minetti et al., 2019. [26] *BAOJ Dentistry*	4 months	36.28%	-	NR	-	14.61%	-
Minetti et al., 2019. [27] *Int J Growth Factors Stem Cells Dent*	6 months	21.51%	-	NR	-	14.61%	-
Ha et al. 2019. [31] *J Korean Dent Sci.*	NO	NR	-	NR	-	NR	-

BM: biomaterial; ATGM: autogenous tooth bone graft; NR: not reported.

**Table 7:** Complications registered when using ATGM.

Author, Year. Journal	Preventive Measures	Nº of Complications	Complications Moment	Type of Complications	Placement Protocol	Nº of OII	Nº of Implant Failures	Survival Rate of OII
Jun et al., 2014. [28] *J Adv Prosthodont.*	AmoxClav, Talniflumato, CHX	NR	NR	NR	Delayed	57	NR	NR
Jeong et al., 2014. [2] *Maxillofac Plast Reconstr Surg.*	NR	NR	NR	NR	NR	NR	NR	NR
Pohl et al., 2016. [30] *Int J Oral Maxillofac Implants.*	NR	No complications	No complications	No complications	Simultaneous and delayed	15	NR	100% (5 years)
Fattouh and Ali, 2018. [29] *EDJ.*	CHX, DicloK, Epidrone, Clinda	2 OII	Intra-surgery	Perforation of Schneider membrane	Delayed	13	0 OII	100% (6 months)
			Post-surgery	Dehiscence				
Minetti et al., 2019. [26] *BAOJ Dentistry*	NR	No complications	No complications	No complications	Delayed	5	NR	NR
Minetti et al., 2019. [27] *Int J Growth Factors Stem Cells Dent*	AmoxClav/Clinda	No complications	No complications	No complications	Delayed	40	1 OII	97.5% (1 year)
Ha et al. 2019. [31] *J Korean Dent Sci.*	NR	6 OII	Intra-surgery	Perforation of Schneider membrane	Simultaneous	67	2 OII	97.01% (4.5 years)
			Post-surgery	Infection; osseointegration failure				

AmoxClav: amoxicillin with clavulanic acid; CHX: chlorhexidine; Clinda: clindamycin; DicloK: potassium diclofenac; NR: not reported; OII: osseointegrated dental implants.
